# Microglia Mediate the Occurrence and Development of Alzheimer’s Disease Through Ligand-Receptor Axis Communication

**DOI:** 10.3389/fnagi.2021.731180

**Published:** 2021-09-20

**Authors:** Chongdong Jian, Lei Wei, Ruikang Mo, Rongjie Li, Lucong Liang, Liechun Chen, Chun Zou, Youshi Meng, Ying Liu, Donghua Zou

**Affiliations:** ^1^Department of Neurology, The Affiliated Hospital of Youjiang Medical University for Nationalities, Baise, China; ^2^Department of Neurology, The Fifth Affiliated Hospital of Guangxi Medical University, Nanning, China; ^3^Department of General Medicine, The Fifth Affiliated Hospital of Guangxi Medical University, Nanning, China; ^4^Department of Geriatrics, The First People’s Hospital of Nanning, Nanning, China

**Keywords:** Alzheimer’s disease, intercellular communication, receptor ligand axis, LASSO, support vector machine

## Abstract

Alzheimer’s disease (AD) is a common neurodegenerative disease. Its onset is insidious and its progression is slow, making diagnosis difficult. In addition, its underlying molecular and cellular mechanisms remain unclear. In this study, clustering analysis was performed on single-cell RNA sequencing (scRNA-seq) data from the prefrontal cortex of 48 AD patients. Each sample module was identified to be a specific AD cell type, eight main brain cell types were identified, and the dysfunctional evolution of each cell type was further explored by pseudo-time analysis. Correlation analysis was then used to explore the relationship between AD cell types and pathological characteristics. In particular, intercellular communication between neurons and glial cells in AD patients was investigated by cell communication analysis. In patients, neuronal cells and glial cells significantly correlated with pathological features, and glial cells appear to play a key role in the development of AD through ligand-receptor axis communication. Marker genes involved in communication between these two cell types were identified using five types of modeling: logistic regression, multivariate logistic regression, least absolute shrinkage and selection operator (LASSO) and support vector machine (SVM). LASSO modeling identified CXCR4, EGFR, MAP4K4, and IGF1R as key genes in this communication. Our results support the idea that microglia play a role in the occurrence and development of AD through ligand-receptor axis communication. In particular, our analyses identify CXCR4, EGFR, MAP4K4, and IGF1R as potential biomarkers and therapeutic targets in AD.

## Introduction

Alzheimer’s disease (AD) is the most common cause of dementia and the most frequent type of progressive neurodegenerative disease. The major clinical characteristics of AD involve cognitive and behavioral dysfunction, and its major pathological feature is unregulated accumulation of amyloid β (Aβ) and intraneuronal neurofibrillary tangles (NFTs) in neurons (Stancu et al., 2014; Hogh, 2017; Zhu et al., 2017). At present, about 47 million people worldwide suffer from dementia, and the number is projected to increase to 131 million by 2,050 (Tiwari et al., 2019).

Due to its insidious onset and slow progression, diagnosis of AD can be difficult (Hogh, 2017). So far, five drugs have been approved by the US Food and Drug Administration (FDA) for the treatment of AD (Cummings et al., 2014). However, these drugs only alleviate disease progression or symptoms, without curing the disease. Thus, there is a critical need to explore the underlying pathogenesis of AD in order to develop therapeutic and even preventive interventions.

AD involves alterations in the interactions between neurons and glial cells (De Strooper and Karran, 2016). Brain damage caused by neuronal death may lead to cognitive decline, memory loss, learning disability and emotional changes (Vasic et al., 2019). Improving the viability of endogenous neuronal progenitor cells (NPCs) can prevent neural injury and degenerative disease (Zou et al., 2016). Activation of the MAPK signaling pathway in neurons may contribute to degenerative neuronal death (Kheiri et al., 2018).

Oligodendrocyte progenitor cells (OPCs), which express the proteoglycan NG2, are the main proliferating cells in the adult central nervous system. OPCs differentiate during postnatal development into myelin-forming oligodendrocytes (de Faria et al., 2019), which wrap around axons in the central nervous system, forming an insulating myelin sheath that assists in signal transmission as well as maintains and protects neuronal function (Nasrabady et al., 2018). During AD development, oligodendrocytes exhibit morphological alterations, deterioration of myelin integrity and axonal destruction (Cai and Xiao, 2016). Apoptosis among oligodendrocytes renders neurons vulnerable to injury and loss.

Microglia, the resident immune cells in the central nervous system, account for approximately 10% of all cells in that system, and they play a subtle and complex role in the development of AD (Lawson et al., 1990; Cameron and Landreth, 2010). Increasing evidence indicates that microglia act as a “double-edged sword,” both helping and harming neurons (Hanisch and Kettenmann, 2007; Sierra et al., 2013). On the one hand, microglia act as scavengers in the brain, clearing substances such as Aβ and thereby preventing the formation of amyloid plaques in the brain. On the other hand, prolonged microglial activation leads to the release of pro-inflammatory cytokines, which trigger a pro-inflammatory cascade that damages and destroys neurons (Perry et al., 2010; Jiang et al., 2012). This cascade involves extensive, complex signaling via chemokines, cytokines and Toll-like receptors (Zhou et al., 2021).

AD development may involve dysregulation of the normally complex yet balanced communication among different cell types in the central nervous system (Vainchtein and Molofsky, 2020). For example, astrocytes and microglia express many cytokines, chemokines and signaling molecules that can activate or destroy neighboring cells, and dysregulation of their production can promote the development of AD (Orre et al., 2014; Ceyzeriat et al., 2018).

Further elucidation of which cell types communicate with one another and of how they communicate may help us understand how AD occurs and progresses. This may then guide the development of novel diagnostic and therapeutic strategies. Toward this end, the present study analyzed single-cell RNA sequencing (scRNA-seq) data from AD brains. Different from previous studies, we provide novel answers and molecular evidences for the cellular communication at the single-cell transcriptome level to mediate the occurrence and development of AD. In particular, microglia play a leading role in the intercellular communication network of AD to regulate the biological signal cascade of target cells through ligand-receptor axis contributing to AD development.

## Materials and Methods

### Data Sources

Postmortem human brain samples were obtained from 48 participants in the Religious Order Study (ROS) or the Rush Memory and Aging Project (MAP), a longitudinal cohort of aging and dementia that included clinical data, detailed post-mortem evaluations, and “omics” tissue profiling (Bennett et al., 2018). Half of these individuals showed substantial Aβ burden and other pathological features of AD, while the other half showed no or extremely low Aβ burden or other pathological features of AD. The prefrontal cortex tissue from these 48 people underwent single-cell RNA sequencing (scRNA-seq), and the data came from Hansruedi Mathys’ group of professors (Mathys et al., 2019).

To identify genes in neuron-glia communication, the following AD datasets from the Gene Expression Omnibus (GEO) database (Barrett et al., 2013) were used as a training set: GSE16759, GSE18309, GSE28146, GSE4757, GSE48350, GSE5281, GSE84422, and GSE9770 on the GPL570 platform. The datasets GSE33000 and GSE44772 on the GPL4372 platform served as a validation set.

### Cell Clustering and Differentially Expressed Genes

The Seurat Package^1^ was used to cluster cells based on scRNA-seq data, and the clusters were visualized using the UMAP package (Becht et al., 2018). In order to define marker genes of clusters, genes differentially expressed between AD patients showing clear pathology and individuals showing no or minimal pathology were determined in each cell type using the “FindAllMarkers” function with the settings min.pct = 0.25 and logfc.threshold = 0.25. Differences associated with *P* < 0.05 were considered significant.

### Pseudo-Time Analysis

We employed the Monocle 2 using variable genes selected by Seurat as the input to determine the evolutionary state of different cell types (Qiu et al., 2017). The gene-cell matrix in the scale of unique molecular identifier (UMI) counts was provided as input to Monocle, and then the “newCellDataSet” function was used to create an object with the parameter expressionFamily = negbinomial.size. After dimension reduction and cell ordering, cell trajectory was inferred using default parameters of Monocle.

### Correlation Between Pathological Features and Differentially Expressed Genes, and Functional Enrichment Analysis

Correlations between pathological features and DEGs were calculated, and those DEGs that were associated with pathological features were screened for functional enrichment based on Kyoto Encyclopedia of Genes and Genomes (KEGG) pathway enrichment using the clusterprofiler package (Yu et al., 2012). Results associated with *P* < 0.05 were considered significant.

### Intercellular Communication Analysis

Intercellular communication was performed using the iTALK routine within the R package. The receptor ligand interaction was determined using the STRING database of protein-protein interactions (Szklarczyk et al., 2017).

### Construction of a Diagnostic Model and Analysis of Receiver Operating Characteristic Curves

A logistic regression model was constructed based on the optimal features of DEGs that correlated with AD pathology. Subsequent multivariate logistic regression was performed using genes that were significant by logistic regression. In addition, least absolute shrinkage and selection operator (LASSO) logistic regression was performed using the “glmnet” routine in R (project.org/package=glmnet). The LASSO model that was able to group samples based on the smallest number of DEGs was selected, and the output was analyzed using the “plot” function. The “tune” function in R (e1071) was used to optimize gamma values and generational values via 10-fold cross validation from an initial range from 10^–20^ to 10^20^ (Jiang et al., 2020). Finally, a support vector machine (SVM) model was also constructed.

To assess the diagnostic efficacy of the three models, we performed ROC curve analysis using the pROC package (Robin et al., 2011), allowing calculation of the area under the ROC curve (AUC).

### Molecular Docking of Ligands Into Receptors

PDB files of key candidate ligands and receptors were downloaded from the Protein Database^2^ (Burley et al., 2017), and molecular docking studies were performed using Hex8.0.0 software (Macindoe et al., 2010). Docking models were visualized using Pymol software (Mooers, 2020).

## Results

### Single-Cell Transcriptomic Landscape in Brain Tissue From Alzheimer’s Disease Patients and Controls

**FIGURE 1:**
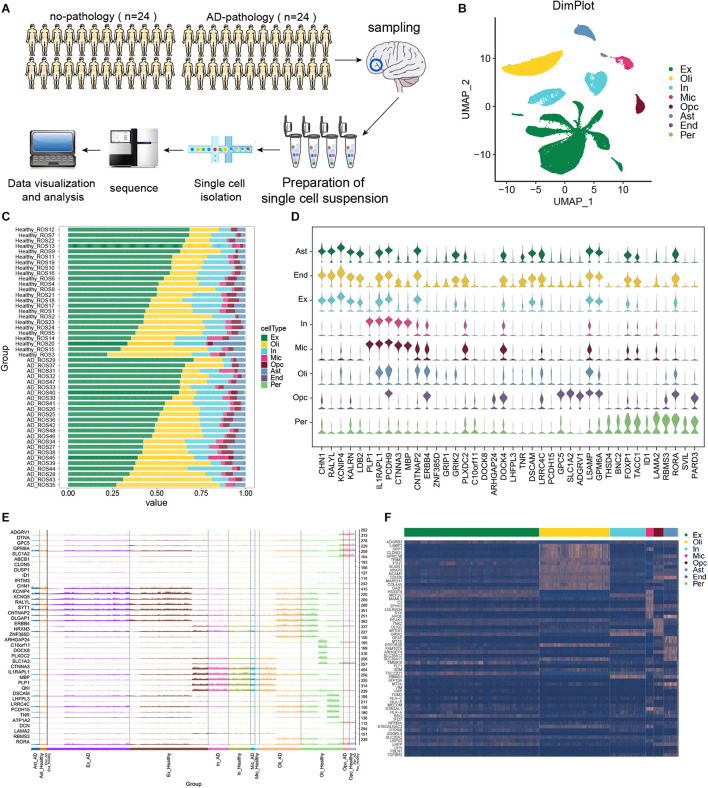
Single-cell transcriptomic landscape of brain tissue from individuals showing strong Alzheimer’s disease pathology (“AD-pathology”) or weak or no pathology (“no-pathology”). **(A)** Study cohort and sample analysis. **(B)** Annotated single-cell profiles of the samples. Eight cell types were identified: excitatory neurons (Ex), oligodendrocytes (Oli), inhibitory neurons (In), microglia (Mic), oligodendrocyte progenitor cells (Opc), astrocytes (Ast), endothelial cells (End) and pericytes (Per). **(C)** Variations in cellular components. **(D)** Expression of the top 5 marker genes for each cell type in no-pathology patients. **(E)** Differences in expression of the top 5 marker genes in each cell type between AD-pathology and no-pathology individuals. High peaks indicate higher expression, while low peaks indicate lower expression. **(F)** Heat map showing marker genes specifically expressed in each cell type in the AD-pathology group.

In this study, scRNA-seq data were analyzed from the prefrontal cortex of individuals with clear AD pathology or with no or minimal pathology. The experimental design flow chart is shown in Figure 1A. These cells were divided into eight types (Figure 1B): excitatory neurons (Ex), oligodendrocytes (Oli), inhibitory neurons (In), microglia (Mic), OPCs, astrocytes (Ast), endothelial cells (End), and pericytes (Per). The compositions of different cell types in each sample are shown in Figure 1C. We found that in individuals showing no or little AD pathology, IL1RAPL1, PCDH9, CNTNAP2, LSMP, and RORA could serve as cellular markers in most cell types (Figure 1D). Figure 1E shows the marker gene expression patterns in each cell type that were shared between individuals with AD or with no or little AD pathology.

To further explore cell specificity between individuals with or without strong AD pathology, potential marker genes of AD pathology were extracted, genes overlapping with common marker genes were removed, and those specifically expressed in individuals with strong AD pathology were identified (Figure 1F).

### Evolution of Expression Dysregulation During Alzheimer’s Disease Progression

**FIGURE 2:**
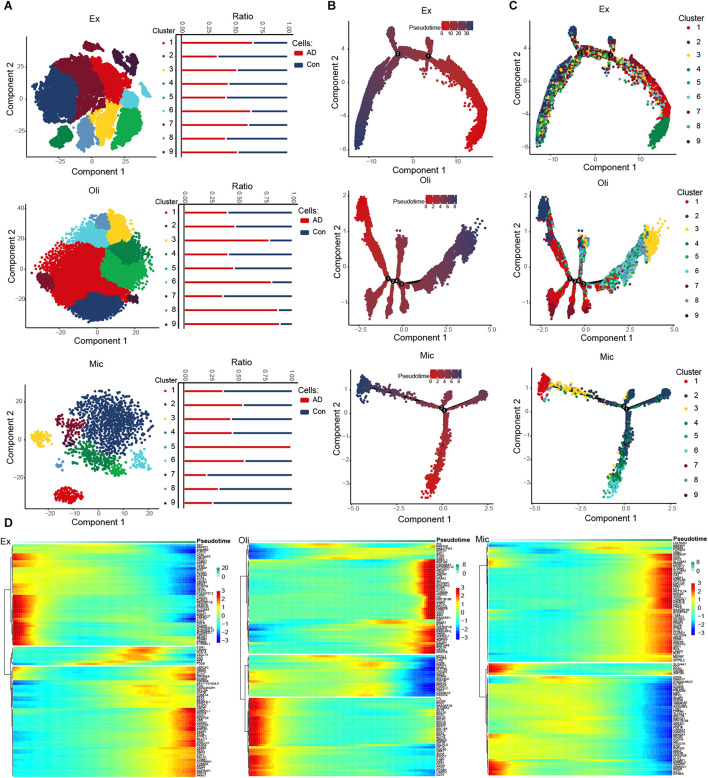
Evolution of expression dysregulation in specific cell types during the course of AD. **(A)** T-distribution stochastic neighbor embedding (T-SNE) plot of excitatory neurons, oligodendrocytes and microglia cells showing nine clusters. The bar chart shows the abundance of cell subsets in individuals showing strong or no/weak AD pathology. **(B)** Pseudotime analysis of excitatory neurons, oligodendrocytes and microglia. Darker color indicates longer pseudotime. **(C)** Distribution of cells in different clusters in the trajectories of excitatory neurons, oligodendrocytes and microglia. **(D)** Expression distribution and heat map of genes as a function of pseudotime. Genes showing similar expression trends fell into distinct clusters.

We identified nine clusters in excitatory neurons, oligodendrocytes and microglia, and we also observed the abundance of these cell subsets in AD and control groups (Figure 2A). Interestingly, cluster 5 abundance was highest in microglia. Then a trajectory was constructed for each cell type using Monocle. We found that the various cell types followed different trajectories. Cell changes over pseudo-time were shown in Figure 2B, and different clusters were enriched along different paths (Figure 2C). We explored DEGs across pseudo-time in different cell types (Figure 2D and Supplementary Figure 1).

### Pathological Process of Alzheimer’s Disease at the Single-Cell Level

**FIGURE 3:**
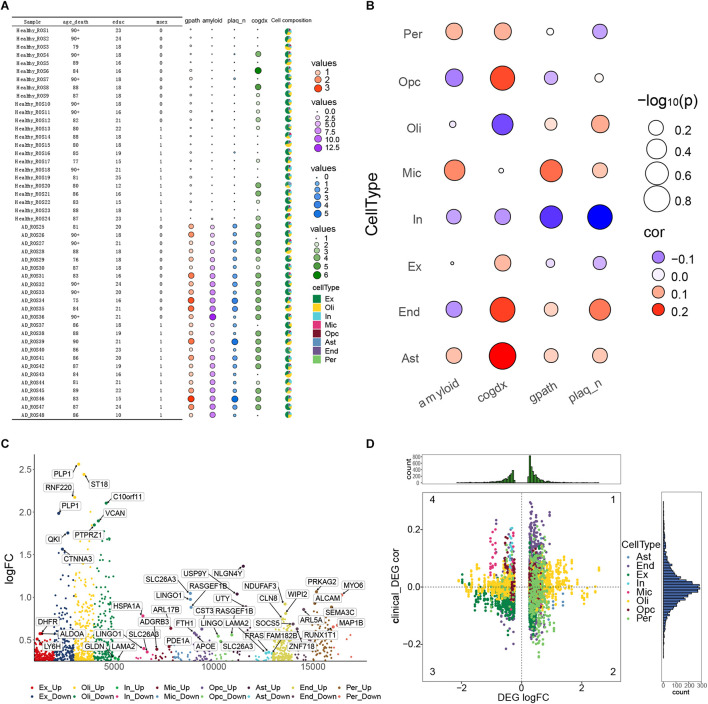
Alzheimer’s disease pathological processes at the single-cell level. **(A)** Clinical data, pathological features and cell types of 48 samples were analyzed. Variables included age_death, defined as age at death; education, years of education; msex, self-reported sexual orientation; “1,” male sex; “0,” female sex; gpath, global AD pathology burden; plaq_n, neuritic plaque burden; amyloid, overall amyloid level; and cogdx, cognitive state at death. **(B)** Bubble heatmaps demonstrating cell abundances in individuals with strong AD pathology (gpath, amyloid, plaq_n, Cogdx). **(C)** Manhattan map showing genes within each cell type that were differentially expressed between the AD-pathology and no-pathology groups. **(D)** Quadrant diagram showing the correlation between pathological characteristics and differentially expressed genes. The ordinate represents correlation coefficient, the abscissa represents log [fold change (FC)], and color represents cell type.

In order to understand the pathological changes during the process of AD, we analyzed the clinical data, pathological features and cell types in all 48 samples. We found that compared with individuals showing weak or no AD pathology, those showing strong pathology showed significantly higher global AD pathology burden (gpath), overall amyloid level (amyloid) and neuritic plaque burden (plaq_n) (Figure 3A). In contrast, the two groups of individuals did not differ significantly in cognitive state at death (cogdx). Then we calculated the correlation between the abundance of each cell type in each sample and the four pathological characteristics (amyloid, cogdx, gpath, plaq_n). We found that microglia significantly correlated positively with amyloid and the gpath. OPCs correlated negatively with amyloid and positively with cogdx. Oligodendrocytes correlated positively with cogdx, while inhibitory neurons correlated negatively with gpath and plaq_n (Figure 3B).

In order to achieve a broad description of the AD ecosystem, we identified DEGs in each cell type between the two groups of individuals (Figure 3C), as well as their correlations with pathological features (Figure 3D). Those DEGs showing a correlation with such features were analyzed further as described below.

### Microglia Mediate Alzheimer’s Disease Occurrence and Development

**FIGURE 4:**
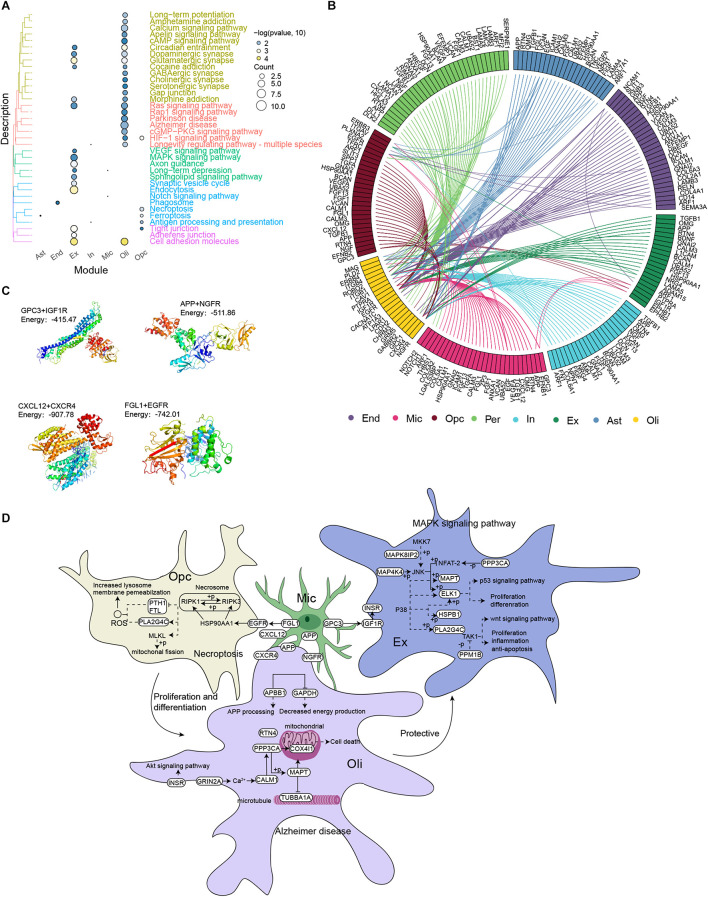
Microglia mediate the occurrence and development of Alzheimer’s disease. **(A)** Clustering of differentially expressed genes in each cell type according to KEGG pathways. **(B)** Integrated regulatory network diagram showing interactions between receptors and ligands encoded by pathway genes in AD. **(C)** Molecular docking between GPC3 and IGF1R, APP and NGFR, CXCL12 and CXCR4, as well as FGL1 and EGFR. Units: kJ per mol. **(D)** Microglia are postulated to communicate with neurons through the GPC3-IGF1R axis, activating the MAPK signaling pathway, promoting neuronal apoptosis and thus mediating the occurrence and development of AD. Microglia may target oligodendrocytes through the APP-NGFR and CXCL12-CXCR4 axes, activating AD signaling pathways and thereby promoting disease occurrence and development. Microglia may target oligodendrocyte progenitor cells through the FGL1-EGFR axis to activate the necrotic apoptotic pathway, triggering the death of oligodendrocytes and rendering neurons vulnerable to injury. This, in turn, contributes to AD occurrence and development.

Among the KEGG pathways enriched with DEGs in each cell type (Figure 4A), the pathways MAPK signaling, AD, necroptosis, ferroptosis and Notch signaling attracted our attention, because they have been related to AD progression. We analyzed intercellular communication in the two groups of individuals in order to explore differentially expressed ligands and receptors in different cell types. This allowed us to construct a comprehensive regulatory network that may contribute to AD (Figure 4B). The iTALK analysis identified significant differences in the abundance of ligand-receptor pairs. Microglia targeted excitatory neurons through the GPC3-IGF1R axis, oligodendrocytes through the APP-NGFR and CXCL12-CXCR4 axes and OPCs through the FGL1-EGFR axis. Molecular docking studies predicted negative docking energies for GPC3 and IGF1R, APP and NGFR, CXCL12 and CXCR4, as well as FGL1 and EGFR, which suggests that such ligand-receptor complexes can form *in vivo* (Figure 4C).

Based on these results, we postulate that microglia communicate directly with neurons through the GPC3-IGF1R axis, which then activates the MAPK signaling pathway to promote neuronal apoptosis and thereby mediate the development and progression of AD. In contrast, microglia target oligodendrocytes through the APP-NGFR and CXCL12-CXCR4 axes to activate the AD pathway and thereby promote the development and progression of the disease. In addition, microglia target OPCs through the FGL1-EGFR axis to activate necrotic apoptosis, which reduces the abundance and activity of oligodendrocytes and thereby renders neurons vulnerable to injury, contributing to AD development (Figure 4D).

### Hub Genes as Biomarkers for Alzheimer’s Disease Diagnosis

**FIGURE 5:**
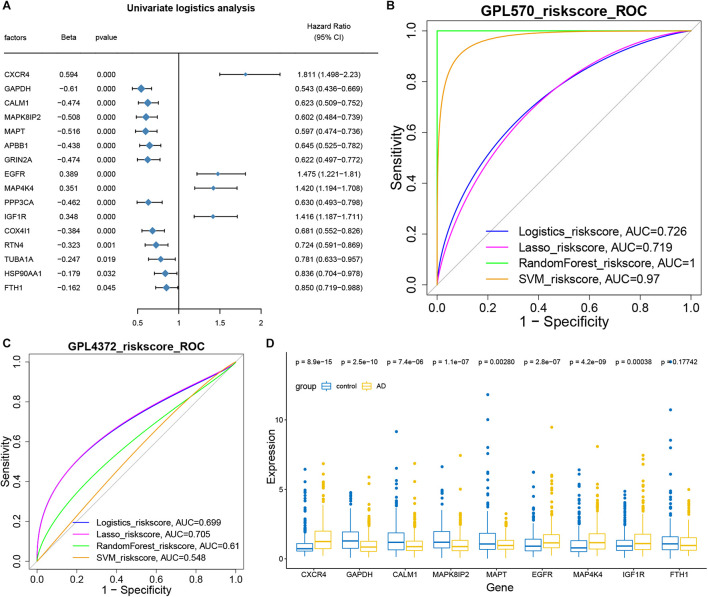
Hub genes as biomarkers for the diagnosis of Alzheimer’s disease. **(A)** Forest map showing single-factor logistic diagnostic efficacy of potential mechanistic genes. CXCR4, EGFR, MAP4K4, and IGF1R emerged as disease risk factors. **(B)** AUCs for multivariate logistic, LASSO, random forest, and SVM modeling. All AUCs were more than 0.5 and therefore considered significant. **(C)** AUCs for the four models based on GSE33000 and GSE44772 datasets on the GPL4372 platform. AUCs were more than 0.5. **(D)** Box diagram showing the expression of core genes (model genes) in individuals with strong AD pathology or no/weak pathology.

The hub genes for intercellular communication involved in the progression of AD development were extracted for univariate logistic analysis, and 16 genes with a significant *P*-value were obtained (Figure 5A). These 16 genes were then used to conduct multi-factor logistics modeling, LASSO modeling, random forest modeling, and SVM modeling. These models gave respective AUCs of 0.726, 0.719, 1.000, and 0.970 for discriminating individuals with severe or minimal AD pathology (Figure 5B).

Then we used GSE33000 and GSE44772 datasets of the GPL4372 platform to verify the four models (Figure 5C). AUCs were 0.699 for logistic modeling, 0.705 for LASSO modeling, 0.61 for random forest modeling, and 0.548 for SVM modeling. Since the LASSO model showed AUC > 0.7 for both the training and validation sets, it was selected as the optimal model. Nine characteristic genes obtained from the LASSO modeling were extracted, and CXCR4, EGFR, MAP4K4, and IGF1R were found to be expressed at higher levels in individuals with AD than in the control group (Figure 5D). Thus, these four genes may be useful in the diagnosis of AD.

## Discussion

AD is one of the most common and devastating aging-related neurodegenerative disorders. Previous studies on AD have focused mainly on data from single cells, but few studies have examined intercellular communication affecting biological processes within cells (Hansen et al., 2018; Wang and Colonna, 2019). For example, studies on AD pathophysiology have focused on gene transcription (Mantzavinos and Alexiou, 2017; Lashley et al., 2018), showing that transcription factors regulate disease processes in AD (Zou C. et al., 2019). Other studies have shown that the microRNA miR-34a plays an important role in AD pathology, mainly by inhibiting the amyloidogenic processing of APP (Jian et al., 2017). Here we report single-cell transcriptomics for AD-related pathology based on a cohort of 48 individuals showing strong disease features or no or minimal pathology. Of eight major cell types in the brain, we identified microglia, OPCs, oligodendrocytes, and excitatory neurons as playing crucial roles in the progression of AD. Neuronal and glial cells correlated with several pathognomonic features of AD: global AD pathology burden, neuritic plaque burden, overall amyloid level, and cognitive state at death. Most hallmark genes were expressed specifically in individuals with strong AD pathology. Our results are consistent with the idea that AD onset and progression involve alterations not in a single gene but in intercellular connections.

The ligand-receptor axis is a fundamental means of communication between cells. Ligand binding to specific cell surface receptors can initiate intracellular signaling cascades that lead to various cellular responses (Fafian-Labora and O’Loghlen, 2020). The receptor-ligand axis can mediate the development of diseases (Allard et al., 2012). For instance, many ligand-receptor signaling patterns have been described between hepatocytes, illustrating the crucial role of intrahepatic crosstalk in tissue homeostasis and injury response (Krenkel and Tacke, 2017). Our data indicate extensive association of microglia with neurons, oligodendrocytes, and OPCs in AD. This association may involve the p38 MAPK pathway, since such signaling has been shown to mediate neuroinflammation triggered by microglia and astrocytes, and it has also been associated with autophagy-type AD (Zou D. et al., 2019). Studies have shown that the GLP-1 ligand-receptor axis is involved not only in AD but also in Parkinson’s and Huntington’s diseases (Janssens et al., 2014). The present study extends this literature by clarifying additional ligand-receptor axes involved in AD.

In the present work, the marker genes CXCR4, EGFR, MAP4K4, and IGF1R were highly expressed in the prefrontal cortex of individuals with strong AD pathology. CXCR4 has previously been shown to regulate neuronal firing and neuron/glia communication (Bezzi et al., 2001). In recent studies, EGFR has been validated as a possible target in the treatment of AD (Tsuji et al., 2021). Development of MAP4 kinase inhibitors can act as motor neuron protectors (Bos et al., 2019). Long-term inhibition of IGF1R signaling can attenuate AD progression and promote neuroprotection (George et al., 2017). These studies and our results argue strongly that these four genes may serve as biomarkers for AD diagnosis.

Our results should be interpreted with caution in light of some limitations. Overfitting is possible, given the strong performance of the model on the training set (AUC = 0.719), yet the model worked well with the validation set (AUC = 0.705), suggesting robustness and repeatability. Nevertheless, our results were obtained entirely through bioinformatic analysis, so they should be verified and extended in functional biochemical studies.

## Conclusion

Our study suggests that microglia may mediate the occurrence and development of AD through ligand-receptor axis communication. We also identified CXCR4, EGFR, MAP4K4, and IGF1R as potential biomarkers of the disease and as candidate therapeutic targets.

## Data Availability Statement

The datasets presented in this study can be found in online repositories. The names of the repository/repositories and accession number(s) can be found in the article/Supplementary Material.

## Author Contributions

CJ, LW, RM, YL, and DZ conceived and designed the study. LW, RL, LC, LL, CZ, and YM collected the data. All authors analyzed the data, prepared figures and tables, wrote the manuscript, reviewed the manuscript, and approved its submission.

## Conflict of Interest

The authors declare that the research was conducted in the absence of any commercial or financial relationships that could be construed as a potential conflict of interest.

## Publisher’s Note

All claims expressed in this article are solely those of the authors and do not necessarily represent those of their affiliated organizations, or those of the publisher, the editors and the reviewers. Any product that may be evaluated in this article, or claim that may be made by its manufacturer, is not guaranteed or endorsed by the publisher.

## Footnotes

1
http://satijalab.org/seurat/

2
www.rcsb.org

## Supplementary Material

Supplementary Figure 1 Evolution of expression dysregulation in relevant cell types during the course of AD. **(A)** T-distribution stochastic neighbor embedding (T-SNE) plot of inhibitory neurons and astrocytes, showing nine clusters. The bar chart shows the abundance of cell subsets in individuals showing strong or no/weak AD pathology. **(B)** Pseudotime of inhibitory neurons and astrocytes. Darker color indicates longer pseudotime. **(C)** Distribution of cells in different clusters in the trajectories of inhibitory neurons and astrocytes. **(D)** Expression distribution and heat map of genes as a function of pseudotime. Genes showing similar expression trends fell into distinct clusters.

## Abbreviations

AD: Alzheimer’s disease
A β: hyperphosphorylated amyloid β
Ast: astrocytes
AUC: area under the receiver operating characteristic curve
End: endothelial cells
Ex: excitatory neuron
GEO: Gene Expression Omnibus
In: inhibitory neuron
KEGG: Kyoto Encyclopedia of Genes and Genomes
LASSO: least absolute shrinkage and selection operator
Mic: microglia
Oli: oligodendrocyte
OPC: oligodendrocyte progenitor cell
Per: pericytes
ROC: receiver operating characteristic
ROSMAP: Religious Orders Study
scRNA-seq: single-cell RNA sequencing
SVM: support vector machine
T-SNE: T-distribution stochastic neighbor embedding
UMAP: uniform manifold approximation and projection
UMI: unique molecular identifier.

## References

[B1] Allard J. F. Dushek O. Coombs D. van der Merwe P. A. (2012). Mechanical modulation of receptor-ligand interactions at cell-cell interfaces. *Biophys. J.* 102 1265–1273. 10.1016/j.bpj.2012.02.006 22455909 PMC3309404

[B2] Barrett T. Wilhite S. E. Ledoux P. Evangelista C. I Kim F. Tomashevsky M. (2013). NCBI GEO: archive for functional genomics data sets–update. *Nucleic Acids Res.* 41 D991–D995. 10.1093/nar/gks1193 23193258 PMC3531084

[B3] Becht E. McInnes L. Healy J. Dutertre C. A. I Kwok W. H. Ng L. G. (2018). Dimensionality reduction for visualizing single-cell data using UMAP. *Nat. Biotechnol. [Online ahead of print]* 10.1038/nbt.4314 30531897

[B4] Bennett D. A. Buchman A. S. Boyle P. A. Barnes L. L. Wilson R. S. Schneider J. A. (2018). Religious orders study and rush memory and aging project. *J. Alzheimers Dis.* 64 S161–S189. 10.3233/JAD-179939 29865057 PMC6380522

[B5] Bezzi P. Domercq M. Brambilla L. Galli R. Schols D. De Clercq E. (2001). CXCR4-activated astrocyte glutamate release via TNFalpha: amplification by microglia triggers neurotoxicity. *Nat. Neurosci.* 4 702–710. 10.1038/89490 11426226

[B6] Bos P. H. Lowry E. R. Costa J. Thams S. Garcia-Diaz A. Zask A. (2019). Development of MAP4 kinase inhibitors as motor neuron-protecting agents. *Cell Chem. Biol.* 26 1703.e37–1715.e37. 10.1016/j.chembiol.2019.10.005 31676236 PMC7253076

[B7] Burley S. K. Berman H. M. Kleywegt G. J. Markley J. L. Nakamura H. Velankar S. (2017). Protein Data Bank (PDB): the single global macromolecular structure archive. *Methods Mol. Biol.* 1607 627–641. 10.1007/978-1-4939-7000-1_26 28573592 PMC5823500

[B8] Cai Z. Xiao M. (2016). Oligodendrocytes and Alzheimer’s disease. *Int. J. Neurosci.* 126 97–104. 10.3109/00207454.2015.1025778 26000818

[B9] Cameron B. Landreth G. E. (2010). Inflammation, microglia, and Alzheimer’s disease. *Neurobiol. Dis.* 37 503–509. 10.1016/j.nbd.2009.10.006 19833208 PMC2823849

[B10] Ceyzeriat K. Ben Haim L. Denizot A. Pommier D. Matos M. Guillemaud O. (2018). Modulation of astrocyte reactivity improves functional deficits in mouse models of Alzheimer’s disease. *Acta Neuropathol. Commun.* 6:104. 10.1186/s40478-018-0606-1 30322407 PMC6190663

[B11] Cummings J. L. Morstorf T. Zhong K. (2014). Alzheimer’s disease drug-development pipeline: few candidates, frequent failures. *Alzheimers Res. Ther.* 6:37. 10.1186/alzrt269 25024750 PMC4095696

[B12] de Faria O. Jr. Dhaunchak A. S. Kamen Y. Roth A. D. Kuhlmann T. Colman D. R. (2019). TMEM10 promotes oligodendrocyte differentiation and is expressed by oligodendrocytes in human remyelinating multiple sclerosis plaques. *Sci. Rep.* 9:3606. 10.1038/s41598-019-40342-x 30837646 PMC6400977

[B13] De Strooper B. Karran E. (2016). The cellular phase of Alzheimer’s disease. *Cell* 164 603–615. 10.1016/j.cell.2015.12.056 26871627

[B14] Fafian-Labora J. A. O’Loghlen A. (2020). Classical and nonclassical intercellular communication in senescence and ageing. *Trends Cell Biol.* 30 628–639. 10.1016/j.tcb.2020.05.003 32505550

[B15] George C. Gontier G. Lacube P. Francois J. C. Holzenberger M. Aid S. (2017). The Alzheimer’s disease transcriptome mimics the neuroprotective signature of IGF-1 receptor-deficient neurons. *Brain* 140 2012–2027. 10.1093/brain/awx132 28595357

[B16] Hanisch U. K. Kettenmann H. (2007). Microglia: active sensor and versatile effector cells in the normal and pathologic brain. *Nat. Neurosci.* 10 1387–1394. 10.1038/nn1997 17965659

[B17] Hansen D. V. Hanson J. E. Sheng M. (2018). Microglia in Alzheimer’s disease. *J. Cell Biol.* 217 459–472. 10.1083/jcb.201709069 29196460 PMC5800817

[B18] Hogh P. (2017). [Alzheimer’s disease]. *Ugeskr Laeger* 179:V09160686. 28330540

[B19] Janssens J. Etienne H. Idriss S. Azmi A. Martin B. Maudsley S. (2014). Systems-Level G protein-coupled receptor therapy across a neurodegenerative continuum by the GLP-1 receptor system. *Front. Endocrinol. (Lausanne)* 5:142. 10.3389/fendo.2014.00142 25225492 PMC4150252

[B20] Jian C. Lu M. Zhang Z. Liu L. Li X. Huang F. (2017). miR-34a knockout attenuates cognitive deficits in APP/PS1 mice through inhibition of the amyloidogenic processing of APP. *Life Sci.* 182 104–111. 10.1016/j.lfs.2017.05.023 28533191

[B21] Jiang H. Gu J. Du J. Qi X. Qian C. Fei B. (2020). A 21gene Support Vector Machine classifier and a 10gene risk score system constructed for patients with gastric cancer. *Mol. Med. Rep.* 21 347–359. 10.3892/mmr.2019.10841 31939629 PMC6896370

[B22] Jiang T. Yu J. T. Tan L. (2012). Novel disease-modifying therapies for Alzheimer’s disease. *J. Alzheimers Dis.* 31 475–492. 10.3233/JAD-2012-120640 22669013

[B23] Kheiri G. Dolatshahi M. Rahmani F. Rezaei N. (2018). Role of p38/MAPKs in Alzheimer’s disease: implications for amyloid beta toxicity targeted therapy. *Rev. Neurosci.* 30 9–30. 10.1515/revneuro-2018-0008 29804103

[B24] Krenkel O. Tacke F. (2017). Liver macrophages in tissue homeostasis and disease. *Nat. Rev. Immunol.* 17 306–321. 10.1038/nri.2017.11 28317925

[B25] Lashley T. Schott J. M. Weston P. Murray C. E. Wellington H. Keshavan A. (2018). Molecular biomarkers of Alzheimer’s disease: progress and prospects. *Dis. Model Mech.* 11:dmm031781. 10.1242/dmm.031781 29739861 PMC5992610

[B26] Lawson L. J. Perry V. H. Dri P. Gordon S. (1990). Heterogeneity in the distribution and morphology of microglia in the normal adult mouse brain. *Neuroscience* 39 151–170. 10.1016/0306-4522(90)90229-w 2089275

[B27] Macindoe G. Mavridis L. Venkatraman V. Devignes M. D. Ritchie D. W. (2010). HexServer: an FFT-based protein docking server powered by graphics processors. *Nucleic Acids Res.* 38 W445–W449. 10.1093/nar/gkq311 20444869 PMC2896144

[B28] Mantzavinos V. Alexiou A. (2017). Biomarkers for Alzheimer’s disease diagnosis. *Curr. Alzheimer Res.* 14 1149–1154. 10.2174/1567205014666170203125942 28164766 PMC5684784

[B29] Mathys H. Davila-Velderrain J. Peng Z. Gao F. Mohammadi S. Young J. Z. (2019). Single-cell transcriptomic analysis of Alzheimer’s disease. *Nature* 570 332–337. 10.1038/s41586-019-1195-2 31042697 PMC6865822

[B30] Mooers B. H. M. (2020). Shortcuts for faster image creation in PyMOL. *Protein Sci.* 29 268–276. 10.1002/pro.3781 31710740 PMC6933860

[B31] Nasrabady S. E. Rizvi B. Goldman J. E. Brickman A. M. (2018). White matter changes in Alzheimer’s disease: a focus on myelin and oligodendrocytes. *Acta Neuropathol. Commun.* 6:22. 10.1186/s40478-018-0515-3 29499767 PMC5834839

[B32] Orre M. Kamphuis W. Osborn L. M. Melief J. Kooijman L. Huitinga I. (2014). Acute isolation and transcriptome characterization of cortical astrocytes and microglia from young and aged mice. *Neurobiol. Aging* 35 1–14. 10.1016/j.neurobiolaging.2013.07.008 23954174

[B33] Perry V. H. Nicoll J. A. Holmes C. (2010). Microglia in neurodegenerative disease. *Nat. Rev. Neurol.* 6 193–201. 10.1038/nrneurol.2010.17 20234358

[B34] Qiu X. Mao Q. Tang Y. Wang L. Chawla R. Pliner H. A. (2017). Reversed graph embedding resolves complex single-cell trajectories. *Nat. Methods* 14 979–982. 10.1038/nmeth.4402 28825705 PMC5764547

[B35] Robin X. Turck N. Hainard A. Tiberti N. Lisacek F. Sanchez J. C. (2011). pROC: an open-source package for R and S+ to analyze and compare ROC curves. *BMC Bioinform.* 12:77. 10.1186/1471-2105-12-77 21414208 PMC3068975

[B36] Sierra A. Abiega O. Shahraz A. Neumann H. (2013). Janus-faced microglia: beneficial and detrimental consequences of microglial phagocytosis. *Front. Cell Neurosci.* 7:6. 10.3389/fncel.2013.00006 23386811 PMC3558702

[B37] Stancu I. C. Vasconcelos B. Terwel D. Dewachter I. (2014). Models of beta-amyloid induced Tau-pathology: the long and “folded” road to understand the mechanism. *Mol. Neurodegener.* 9:51. 10.1186/1750-1326-9-51 25407337 PMC4255655

[B38] Szklarczyk D. Morris J. H. Cook H. Kuhn M. Wyder S. Simonovic M. (2017). The STRING database in 2017: quality-controlled protein-protein association networks, made broadly accessible. *Nucleic Acids Res.* 45 D362–D368. 10.1093/nar/gkw937 27924014 PMC5210637

[B39] Tiwari S. Atluri V. Kaushik A. Yndart A. Nair M. (2019). Alzheimer’s disease: pathogenesis, diagnostics, and therapeutics. *Int. J. Nanomed.* 14 5541–5554. 10.2147/IJN.S200490 31410002 PMC6650620

[B40] Tsuji S. Hase T. Yachie-Kinoshita A. Nishino T. Ghosh S. Kikuchi M. (2021). Artificial intelligence-based computational framework for drug-target prioritization and inference of novel repositionable drugs for Alzheimer’s disease. *Alzheimers Res. Ther.* 13:92. 10.1186/s13195-021-00826-3 33941241 PMC8091739

[B41] Vainchtein I. D. Molofsky A. V. (2020). Astrocytes and microglia: in sickness and in health. *Trends Neurosci.* 43 144–154. 10.1016/j.tins.2020.01.003 32044129 PMC7472912

[B42] Vasic V. Barth K. Schmidt M. H. H. (2019). Neurodegeneration and Neuro-Regeneration-Alzheimer’s disease and stem cell therapy. *Int. J. Mol. Sci.* 20:4272. 10.3390/ijms20174272 31480448 PMC6747457

[B43] Wang S. Colonna M. (2019). Microglia in Alzheimer’s disease: a target for immunotherapy. *J. Leukoc Biol.* 106 219–227. 10.1002/JLB.MR0818-319R 30725482

[B44] Yu G. Wang L. G. Han Y. He Q. Y. (2012). clusterProfiler: an R package for comparing biological themes among gene clusters. *OMICS* 16 284–287. 10.1089/omi.2011.0118 22455463 PMC3339379

[B45] Zhou F. Chen D. Chen G. Liao P. Li R. Nong Q. (2021). Gene set index based on different modules may help differentiate the mechanisms of Alzheimer’s disease and vascular dementia. *Clin. Interv. Aging* 16 451–463. 10.2147/CIA.S297483 33737807 PMC7961151

[B46] Zhu J. B. Tan C. C. Tan L. Yu J. T. (2017). State of play in Alzheimer’s disease genetics. *J. Alzheimers Dis.* 58 631–659. 10.3233/JAD-170062 28505974

[B47] Zou C. Wang J. Huang X. Jian C. Zou D. Li X. (2019). Analysis of transcription factor- and ncRNA-mediated potential pathogenic gene modules in Alzheimer’s disease. *Aging (Albany NY)* 11 6109–6119. 10.18632/aging.102169 31422384 PMC6738443

[B48] Zou D. Li R. Huang X. Chen G. Liu Y. Meng Y. (2019). Identification of molecular correlations of RBM8A with autophagy in Alzheimer’s disease. *Aging (Albany NY)* 11 11673–11685. 10.18632/aging.102571 31816601 PMC6932873

[B49] Zou D. Zhou Y. Liu L. Dong F. Shu T. Zhou Y. (2016). Transient enhancement of proliferation of neural progenitors and impairment of their long-term survival in p25 transgenic mice. *Oncotarget* 7 39148–39161. 10.18632/oncotarget.9834 27283769 PMC5129921

