# Corrigendum: Microglia Mediate the Occurrence and Development of Alzheimer's Disease Through Ligand-Receptor Axis Communication

**DOI:** 10.3389/fnagi.2021.800761

**Published:** 2021-11-19

**Authors:** Chongdong Jian, Lei Wei, Ruikang Mo, Rongjie Li, Lucong Liang, Liechun Chen, Chun Zou, Youshi Meng, Ying Liu, Donghua Zou

**Affiliations:** ^1^Department of Neurology, The Affiliated Hospital of Youjiang Medical University for Nationalities, Baise, China; ^2^Department of Neurology, The Fifth Affiliated Hospital of Guangxi Medical University, Nanning, China; ^3^Department of General Medicine, The Fifth Affiliated Hospital of Guangxi Medical University, Nanning, China; ^4^Department of Geriatrics, The First People's Hospital of Nanning, Nanning, China

**Keywords:** Alzheimer's disease, intercellular communication, receptor ligand axis, LASSO, support vector machine

In the original article, there was an error. **The datasets GSE18309 and GSE9770 were incorrectly written as GSE18039 and GSE977013**.

A correction has been made to ***MATERIALS AND METHODS***, ***Data Sources***, *Paragraph 2*:

**To identify genes in neuron-glia communication, the following AD datasets from the Gene Expression Omnibus (GEO) database (Barrett et al., 2013) were used as a training set: GSE16759, GSE18309, GSE28146, GSE4757, GSE48350, GSE5281, GSE84422, and GSE9770 on the GPL570 platform. The datasets GSE33000 and GSE44772 on the GPL4372 platform served as a validation set**.

The authors apologize for this error and state that this does not change the scientific conclusions of the article in any way. The original article has been updated.

## Publisher's Note

All claims expressed in this article are solely those of the authors and do not necessarily represent those of their affiliated organizations, or those of the publisher, the editors and the reviewers. Any product that may be evaluated in this article, or claim that may be made by its manufacturer, is not guaranteed or endorsed by the publisher.

