# Supplementary figures and images for: Microglia Mediate the Occurrence and Development of Alzheimer’s Disease Through Ligand-Receptor Axis Communication
Source: Front Aging Neurosci. 2021 Sep 20;13:731180. doi: 10.3389/fnagi.2021.731180 (PMC8488208; doi:10.3389/fnagi.2021.731180)

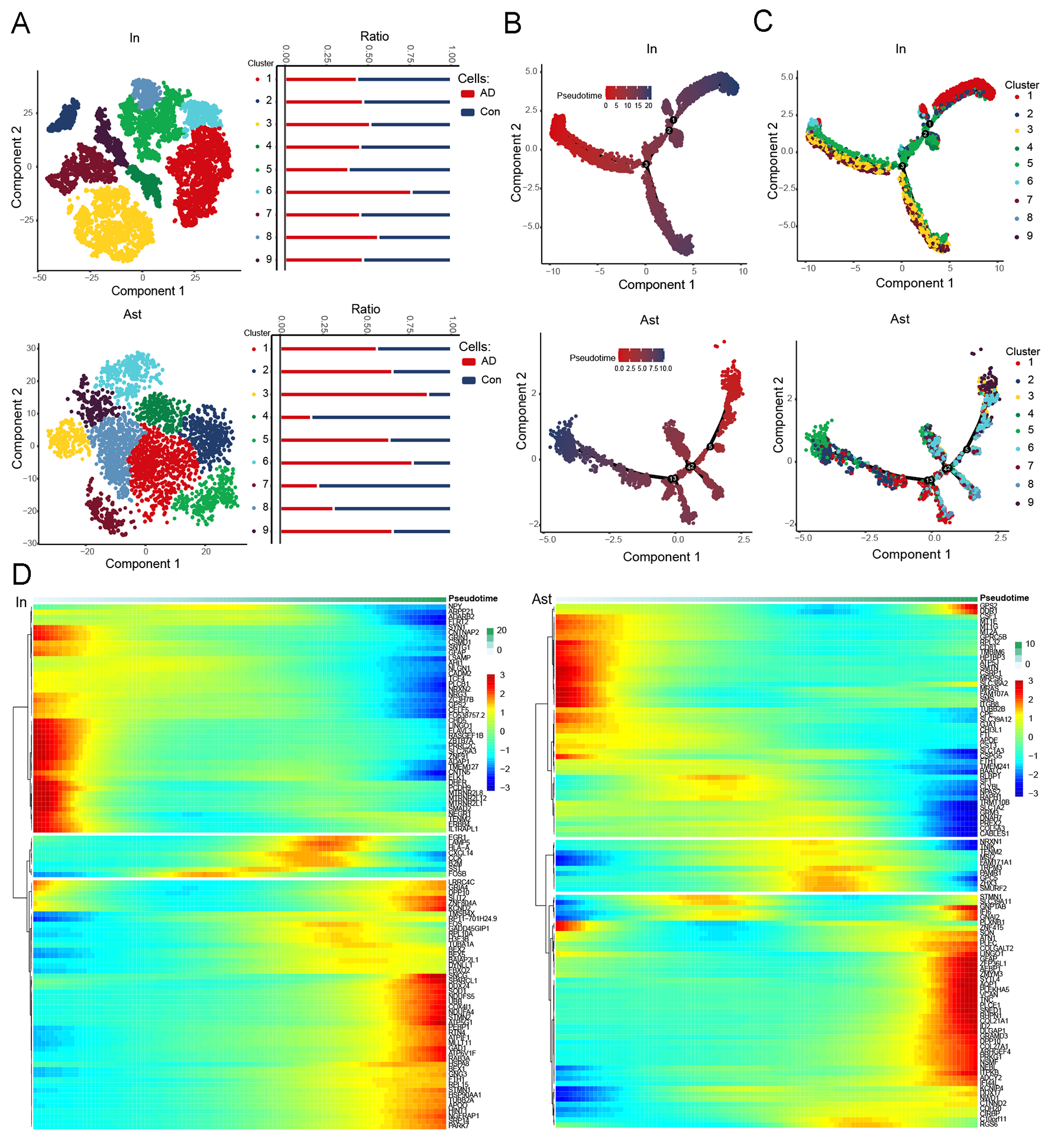

Supplement: Supplementary Figure 1 — Evolution of expression dysregulation in relevant cell types during the course of AD. (A) T-distribution stochastic neighbor embedding (T-SNE) plot of inhibitory neurons and astrocytes, showing nine clusters. The bar chart shows the abundance of cell subsets in individuals showing strong or no/weak AD pathology. (B) Pseudotime of inhibitory neurons and astrocytes. Darker color indicates longer pseudotime. (C) Distribution of cells in different clusters in the trajectories of inhibitory neurons and astrocytes. (D) Expression distribution and heat map of genes as a function of pseudotime. Genes showing similar expression trends fell into distinct clusters. [file Image_1.TIF]
